# Coenzyme Q10 supplementation enhances HSP70 gene expression and preserves mitochondrial function in cryopreserved Peranakan Ettawa goat spermatozoa

**DOI:** 10.14202/vetworld.2026.3443-3456

**Published:** 2026-08-08

**Authors:** Yudit Oktanella, Imam Mustofa, Suherni Susilowati, Widjiati Widjiati, Tri Wahyu Suprayogi, Yayuk Kholifah

**Affiliations:** 1 Doctoral Program of Veterinary Science, Faculty of Veterinary Medicine, Universitas Airlangga, Surabaya, Indonesia.; 2 Department of Veterinary Reproduction, Faculty of Veterinary Medicine, Universitas Brawijaya, Malang, East Java, Indonesia.; 3 Division of Veterinary Reproduction, Faculty of Veterinary Medicine, Universitas Airlangga, Surabaya, Indonesia.; 4 Division of Veterinary Embryology, Faculty of Veterinary Medicine, Universitas Airlangga, Surabaya, Indonesia.; 5 National Artificial Insemination Center (BBIB) Singosari, Malang, East Java, Indonesia.

**Keywords:** antioxidant, coenzyme Q10, cryopreservation, goat spermatozoa, HSP70, mitochondrial membrane potential, oxidative stress, sperm viability

## Abstract

**Background and Aim:**

Cryopreservation is essential for the long-term preservation and dissemination of superior goat genetics but induces oxidative, osmotic, and thermal stress that compromises sperm quality and fertilizing potential. Mitochondrial dysfunction, lipid peroxidation, and impaired cellular stress responses are major contributors to cryoinjury. Coenzyme Q10 (CoQ10), a mitochondrial electron carrier and potent lipid-soluble antioxidant, has been proposed as a cryoprotective supplement; however, its effects on heat shock protein 70 (*HSP70*) gene expression and mitochondrial function in cryopreserved Peranakan Ettawa (PE) goat spermatozoa remain insufficiently characterized. This study evaluated the effects of graded CoQ10 supplementation in Extender A on post-thaw *HSP70* gene expression, mitochondrial membrane potential (MMP), lipid peroxidation, and sperm viability.

**Materials and Methods:**

Semen was collected from one clinically healthy 4-year-old PE buck, and eight independent ejaculates were processed using a split-ejaculate design. Each ejaculate was divided into five treatment groups supplemented with CoQ10 at 0 (C0), 5 (C1), 10 (C2), 20 (C3), and 40 mg/dL (C4). Following cryopreservation and thawing, *HSP70* gene expression was quantified using reverse-transcription quantitative polymerase chain reaction (RT-qPCR) with Glyceraldehyde-3-phosphate dehydrogenase (*GAPDH*) as the reference gene. MMP was assessed using rhodamine 123 staining and confocal microscopy, lipid peroxidation was determined by measuring malondialdehyde (MDA) concentrations, and sperm viability was evaluated using eosin–nigrosine staining. Data were analyzed using one-way analysis of variance followed by Tukey or Games-Howell post hoc tests, with statistical significance set at p < 0.05.

**Results:**

CoQ10 supplementation significantly improved all evaluated post-thaw parameters (p < 0.001). The C4 group exhibited the highest *HSP70* expression (3.19 ± 0.08), representing approximately a 6.5-fold increase over the control, together with the highest MMP (56.91 ± 4.21%) and sperm viability (64.37 ± 6.58%). In contrast, the lowest MDA concentration was observed in C2 (8.20 ± 3.27 ng/µL), whereas the control group showed the highest lipid peroxidation (28.80 ± 1.38 ng/µL). These findings indicate a dose-dependent but non-linear antioxidant response, with higher CoQ10 concentrations preferentially supporting mitochondrial integrity and cellular stress adaptation rather than maximally suppressing lipid peroxidation.

**Conclusion:**

Supplementation of Extender A with CoQ10 effectively enhanced post-thaw quality of PE goat spermatozoa by preserving mitochondrial function, improving sperm viability, and upregulating *HSP70* gene expression. Although 10 mg/dL most effectively reduced lipid peroxidation, 40 mg/dL provided the most favorable overall cryoprotective profile and represents a promising candidate dose for further optimization of goat semen cryopreservation protocols. Future studies involving multiple bucks, fertility trials, and mechanistic investigations at the protein level are warranted to validate these findings and facilitate the practical application of CoQ10 in artificial insemination programs.

## INTRODUCTION

Goats (*Capra hircus*) play a pivotal role in food security, household income generation, and the livelihoods of smallholder farmers in many developing countries, including Indonesia [1]. Artificial insemination (AI) is an effective reproductive biotechnology for accelerating genetic improvement and increasing productivity in goat populations. However, the success of AI programs depends largely on the availability of frozen semen with consistently high post-thaw quality and fertilizing capacity [2]. Cryopreservation inevitably exposes spermatozoa to osmotic imbalance, ice-crystal formation, temperature fluctuations, and oxidative stress, all of which impair sperm motility, membrane integrity, viability, and fertilizing potential [3, 4]. Compared with several other domestic ruminants, goat spermatozoa are particularly susceptible to cryoinjury, resulting in reduced post-thaw survival and limiting the efficiency of large-scale AI programs [5, 6]. The Peranakan Ettawa (PE) goat is an economically important dual-purpose breed in Indonesia; therefore, improving semen cryopreservation protocols for this breed has substantial implications for national breeding and genetic improvement programs.

The freeze–thaw process induces excessive production of reactive oxygen species (ROS), overwhelming the limited endogenous antioxidant defense system of spermatozoa [7, 8]. Oxidative stress primarily disrupts mitochondrial function, commonly manifested as collapse of the mitochondrial membrane potential (MMP), ultimately reducing adenosine triphosphate (ATP) production and sperm motility [9]. Progressive mitochondrial dysfunction may also promote apoptosis-like changes via dysregulated mitochondrial membrane permeability, resulting in membrane damage, impaired cellular metabolism, and reduced sperm survival [10–13]. In response to thermal and oxidative stress, heat shock protein 70 (*HSP70*) functions as a molecular chaperone that stabilizes protein conformation, facilitates protein refolding, and maintains cellular homeostasis [14, 15]. Members of the heat shock protein (HSP) family have been associated with enhanced cryotolerance and improved post-thaw sperm quality in several animal species [16, 17]. This protective mechanism is particularly important because the sperm midpiece contains a highly specialized mitochondrial sheath that generates the ATP required for flagellar movement and fertilization competence [18–21]. Consequently, the preservation of mitochondrial integrity, together with the activation of cellular stress-response mechanisms, is considered essential for maintaining post-thaw sperm function.

Coenzyme Q10 (CoQ10) is a lipid-soluble quinone located within the inner mitochondrial membrane, where it functions as an electron carrier in the mitochondrial respiratory chain while simultaneously serving as a redox-active antioxidant [22]. Previous studies have demonstrated that CoQ10 supplementation in semen extenders improves several post-thaw sperm characteristics and modulates the expression of mitochondrial-related genes in goats and other livestock species [23, 24]. Nevertheless, the mechanisms underlying these beneficial effects remain incompletely understood, particularly regarding interactions among CoQ10 supplementation, mitochondrial preservation, and cellular stress-response pathways. Because oxidative stress and mitochondrial dysfunction are closely interconnected during cryopreservation, interventions that simultaneously preserve mitochondrial function and enhance stress-response capacity may provide superior cryoprotection compared with antioxidant activity alone.

Despite increasing evidence supporting the beneficial effects of CoQ10 during semen cryopreservation, several important knowledge gaps remain. Most previous studies have primarily evaluated conventional semen-quality parameters, such as motility, viability, membrane integrity, and oxidative stress biomarkers, whereas relatively few have investigated the molecular mechanisms underlying CoQ10-mediated cryoprotection. In particular, the relationship between CoQ10 supplementation and *HSP70* gene expression, as well as mitochondrial function, has not been comprehensively explored in cryopreserved PE goat spermatozoa. Furthermore, information on the optimal CoQ10 concentration that can simultaneously enhance mitochondrial integrity, reduce lipid peroxidation, promote *HSP70* expression, and improve post-thaw sperm viability remains limited. Addressing these knowledge gaps would provide mechanistic insights into the role of CoQ10 during sperm cryopreservation and facilitate the development of more effective semen-preservation strategies for AI programs.

This study aimed to evaluate the effects of graded CoQ10 supplementation (0–40 mg/dL) in Extender A on the post-thaw quality of cryopreserved PE goat spermatozoa by assessing *HSP70* transcript abundance, MMP, malondialdehyde (MDA) concentration, and sperm viability. We hypothesized that CoQ10 supplementation would preserve mitochondrial function, reduce oxidative damage, upregulate *HSP70* expression, and consequently improve post-thaw sperm viability. The study further sought to identify the CoQ10 concentration that provides the most favorable overall cryoprotective profile for PE goat semen.

## MATERIALS AND METHODS

### Ethical approval

The experimental protocol involving animals was reviewed and approved by the Institutional Animal Ethics Committee, Faculty of Veterinary Medicine, Universitas Brawijaya, Malang, Indonesia (Ethical Clearance No. 82-KEP-FKHUB-2026). All animal procedures were conducted in accordance with the institutional guidelines for the care and use of animals for scientific purposes and complied with applicable national regulations governing animal welfare.

The study involved non-invasive semen collection from a clinically healthy Peranakan Ettawa buck maintained under routine husbandry conditions at the National Artificial Insemination Center (Balai Besar Inseminasi Buatan [BBIB]), Singosari, Malang, Indonesia. Semen collection was performed using a locally fabricated artificial vagina by experienced personnel as part of the routine semen collection program, thereby minimizing stress and avoiding unnecessary discomfort to the animal. Throughout the study, animal health and welfare were continuously monitored, and no invasive procedures or treatments beyond standard management practices were performed. All efforts were made to ensure humane handling and to minimize stress during semen collection and sample processing.

### Study period and location

The study was conducted from December 2025 to February 2026 at the National Artificial Insemination Center [BBIB], Singosari, Malang, Indonesia. Semen collection, initial evaluation, processing, cryopreservation, and storage were performed at BBIB. Laboratory analyses were conducted at Universitas Brawijaya, Malang, Indonesia.

### Study design

This exploratory experimental study used a split-ejaculate design to evaluate the effects of graded CoQ10 supplementation in Extender A on post-thaw *HSP70* expression, MMP, MDA concentration, and sperm viability. Each accepted ejaculate was divided into equal aliquots and processed concurrently across all treatment groups to minimize variation among ejaculates. The ejaculate was considered the experimental unit, with eight biological replicates per treatment (n = 8), whereas straws and microscopic fields were regarded as technical subsampling units. A formal a priori power analysis was not performed because the study was designed as an exploratory dose–response experiment using the available semen from a single PE buck.

The cryopreservation workflow, two-step extender system, cooling and freezing schedules, and thawing procedure were adapted from the frozen goat semen protocol described by Oktanella *et al.* [23], with modifications to the CoQ10 dose range and the stage of supplementation. CoQ10 was incorporated into Extender A to provide antioxidant exposure during the initial dilution and cooling stages before glycerolization with Extender B. The treatment groups are presented in Table 1.

**Table 1:** Experimental groups and CoQ10 concentrations in Extender A.

**Group**	**Description**	**CoQ10 concentration in Extender A**
C0	Vehicle-matched control without CoQ10	0 mg/dL
C1	Low-dose CoQ10	5 mg/dL
C2	Moderate-dose CoQ10	10 mg/dL
C3	High-dose CoQ10	20 mg/dL
C4	Very-high-dose CoQ10	40 mg/dL

Note: C0 = Control group; C1 = Low-dose treatment group; C2 = Moderate-dose treatment group; C3 = High-dose treatment group; C4 = Very-high-dose treatment group; CoQ10 = Coenzyme Q10.

### Animals and semen collection

Fresh semen was obtained from one clinically healthy 4-year-old PE buck maintained under routine management at BBIB. Because semen was collected from a single buck, the findings should be interpreted as exploratory and should not be generalized to the entire PE goat population. Eight ejaculates that satisfied the predetermined quality criteria were used as independent biological replicates.

Semen was collected using an artificial vagina. Fresh ejaculates had a pH of 6.8, a volume of 1.2–1.8 mL, a cream-white appearance, a characteristic odor, high density and viscosity, approximately 80% progressive motility, and a sperm concentration of 2.6 × 10⁹ spermatozoa/mL. Only ejaculates that met the screening criteria were processed for cryopreservation.

### Semen extender preparation

A two-step milk–egg yolk extender system comprising Extender A and Extender B was used. Extender A was prepared to a final volume of 100 mL using distilled water, skim milk at 10% weight/volume (w/v), egg yolk at 5% volume/volume (v/v), fructose at 0.75% w/v, penicillin at 1000 IU/mL, and streptomycin at 1 mg/mL. Penicillin and streptomycin were obtained from Gibco (Thermo Fisher Scientific, Waltham, MA, USA).

Skim milk was indirectly heated at 92–95°C for 10 min, then cooled to 37°C. Filtered egg yolk was then added and homogenized using magnetic stirring, followed by the addition of fructose and antibiotics.

CoQ10 (ubiquinone-10, ≥98% purity; Sigma-Aldrich, St. Louis, MO, USA) was initially dissolved in ethanol obtained from Sigma-Aldrich and gradually incorporated into prewarmed Extender A at 37°C under continuous stirring. Final CoQ10 concentrations of 0, 5, 10, 20, and 40 mg/dL were prepared. The ethanol vehicle was matched among treatment groups, and its final concentration was maintained below 0.5% v/v to minimize solvent-related effects. Continuous stirring was used to maintain visual homogeneity. However, high-performance liquid chromatography (HPLC) verification of CoQ10 concentration, stability testing, and measurement of the final extender pH and osmolarity were not performed.

Extender B was prepared from the base extender supplemented with glucose at 2% w/v and glycerol at 12% v/v. Glycerol was obtained from Merck (Darmstadt, Germany). All extenders were prepared aseptically and maintained at appropriate temperatures before use. Pre-freeze motility was assessed using computer-assisted sperm analysis (CASA) as a general quality control measure and was approximately 60% after processing. Because a split-ejaculate allocation was used, separate treatment-specific pre-freeze motility tables were not generated.

### Cryopreservation and thawing procedures

Semen was diluted at 37°C with Extender A according to the assigned treatment group and gradually cooled to 3–5°C over approximately 1 h, corresponding to an estimated cooling rate of approximately 0.55°C/min. After equilibration, glycerolization was performed using Extender B according to the two-step protocol.

The diluted semen was loaded into 0.25-mL French straws, sealed, and arranged on freezing racks. Multiple straws were produced for each treatment–ejaculate combination; however, the number of straws was not treated as the independent sample size. Straws were distributed across different rack positions to minimize positional bias during vapor freezing.

Freezing was performed in liquid nitrogen vapor at a height of 4 cm above the liquid nitrogen surface. The straws were cooled from approximately 4°C to −110°C over 11 min and then plunged into liquid nitrogen at −196°C, where they remained until evaluation. For thawing, frozen straws were immersed in a 37°C water bath (DWBC-6H; PT Maskot Sukses Terus, Tangerang, Indonesia) for 30 s, then wiped dry, opened, and immediately processed for post-thaw analyses.

### Post-thaw assessments

**Sperm viability:** Post-thaw sperm viability was assessed using eosin–nigrosine staining. Thawed semen was mixed in a 1:1 ratio with a staining solution containing 0.67% eosin and 10% nigrosine (Merck). The mixture was smeared onto clean glass slides, air-dried, and examined using an Olympus light microscope (CX21; Olympus Corporation, Tokyo, Japan) at 400×–1000× magnification.

At least 200 spermatozoa per slide were evaluated in randomly selected microscopic fields when sample density permitted. Spermatozoa with eosin-stained heads were classified as nonviable, whereas unstained spermatozoa were classified as viable.

**MMP analysis:** MMP was evaluated using rhodamine 123 (Sigma-Aldrich) and a Leica DMi8 STELLARIS 5 confocal laser scanning microscope (Leica Microsystems, Wetzlar, Germany). Rhodamine 123 is a cationic fluorescent probe that accumulates within mitochondria in a membrane-potential-dependent manner.

Post-thaw semen was washed with phosphate-buffered saline (PBS; HiMedia Laboratories Pvt. Ltd., Thane, Maharashtra, India; pH 7.4), adjusted for microscopic evaluation, and incubated with 10 µg/mL rhodamine 123 in PBS at 37°C in the dark. Samples were then gently washed to remove excess dye and mounted on microscope slides. Laser power, detector gain, and pinhole size were maintained consistently across all treatment groups.

Spermatozoa exhibiting strong fluorescence in the midpiece were classified as having high MMP, whereas those exhibiting weak fluorescence were classified as having low MMP. At least 200 spermatozoa per sample were scored in randomly selected fields whenever possible. Chemical depolarization controls, such as carbonyl cyanide *m*-chlorophenyl hydrazone (CCCP) or valinomycin, were not included. Therefore, the rhodamine 123 results were interpreted as comparative differences in MMP among treatments rather than as absolute measurements of mitochondrial potential.

**MDA assay:** Lipid peroxidation was evaluated by measuring MDA using the thiobarbituric acid (TBA) reaction [25]. Post-thaw aliquots from each treatment group were processed using an identical dilution procedure and reacted with TBA reagent. Absorbance was measured at 532 nm using an iMark Microplate Absorbance Reader (Bio-Rad, Hercules, CA, USA).

MDA concentrations were calculated using an MDA standard curve and expressed as ng/µL. All treatment groups were analyzed using the same procedure to enable valid comparisons among treatments.

**HSP70 gene expression analysis:** Total ribonucleic acid (RNA) was isolated from post-thaw sperm samples using the Direct-zol RNA Extraction Kit (Zymo Research, Irvine, CA, USA) according to the manufacturer’s instructions. Thawed semen was stabilized using DNA/RNA Shield obtained from Zymo Research and lysed using the kit-supplied RNA lysis buffer. Samples were subsequently processed through ethanol-based binding, subjected to on-column deoxyribonuclease (DNase) treatment, washed, and eluted using DNase- and ribonuclease (RNase)-free water.

Potential genomic deoxyribonucleic acid (DNA) contamination and extender-related interference were minimized through washing and DNase treatment. However, independent quantification of residual genomic DNA after extraction was not performed. RNA concentration and purity were assessed using a NanoDrop ND-1000 Spectrophotometer (Thermo Scientific, Waltham, MA, USA). RNA integrity number (RIN) values were not generated because of the characteristically low RNA yield of spermatozoa.

Complementary DNA (cDNA) was synthesized using the iScript cDNA Synthesis Kit obtained from Bio-Rad in a T100 Thermal Cycler obtained from Bio-Rad. The reverse-transcription program comprised incubation at 42°C for 15 min, enzyme inactivation at 95°C for 3 min, and holding at 4°C. The synthesized cDNA was stored at −20°C until analysis.

Reverse-transcription quantitative polymerase chain reaction (RT-qPCR) was performed using a CFX96 Real-Time PCR Detection System (Bio-Rad) with SsoFast EvaGreen Supermix (Bio-Rad). Each reaction contained forward and reverse primers, master mix, nuclease-free water, and cDNA template. The forward and reverse oligonucleotide primers were commercially synthesized by 1st BASE (Axil Scientific Pte. Ltd., Singapore, Singapore) and supplied through PT Genetika Science Indonesia. The primer sequences are presented in Table 2. The amplification conditions comprised an initial denaturation at 95°C for 3 min, followed by 35 cycles at 95°C for 10 s and 60°C for 30 s. Melt-curve analysis was performed after amplification to verify product specificity.

**Figure 1:**
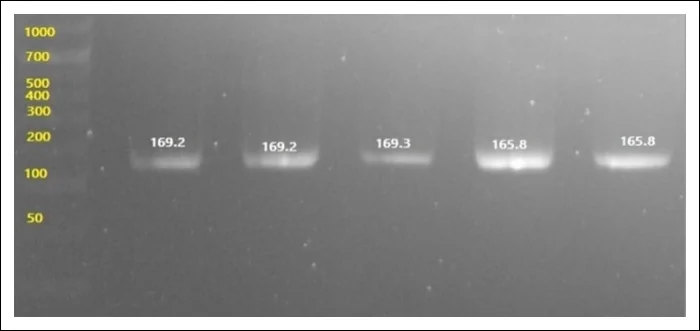
Agarose gel electrophoresis showing the specific amplification of the *HSP70* gene at 167 bp. Lane M represents the DNA ladder. bp = Base pair; DNA = Deoxyribonucleic acid; *HSP70* = Heat shock protein 70.

Primer specificity was initially confirmed using conventional reverse-transcription polymerase chain reaction (RT-PCR), followed by electrophoresis on a 2% agarose gel prepared using UltraPure Agarose (Thermo Fisher Scientific). A 50-bp DNA Step Ladder (Promega Corporation, Madison, WI, USA) was used as the molecular size marker. Electrophoresis revealed a single HSP70 amplicon at the expected size of 167 bp (Figure 1). Relative *HSP70* expression was calculated using the comparative threshold cycle (Ct) method (2^−ΔΔCt), with glyceraldehyde-3-phosphate dehydrogenase (*GAPDH*) as the reference gene [26]. Technical replicates were averaged for each sample.

*GAPDH* was selected because of its common use as a reference gene in sperm gene expression studies. However, the stability of *GAPDH* under the present cryopreservation treatments was not formally validated, and primer amplification efficiencies were not determined. Reporting incorporated the relevant Minimum Information for Publication of Quantitative Real-Time PCR Experiments (MIQE) criteria available for this study, including primer sequences, amplicon sizes, cycling conditions, reference gene selection, and specificity assessment.

**Table 2:** Primer sequences used for amplification of HSP70 and GAPDH.

**Gene**	**Primer sequence (5′–3′)**	**Amplicon size (bp)**	**Accession number**
HSP70	F: CCCACGAAGCAGACGCAGAT	167	XM_002701934.1
	R: CCCAGCAGGTTGTTGTCCCG		
GAPDH	F: AAAGTGGACATCGTCGCCAT	116	NM_001285430.1
	R: CCGTCAGATCCACGACGGAC		

Note: bp = Base pair; F = Forward primer; *GAPDH* = Glyceraldehyde-3-phosphate dehydrogenase; *HSP70* = Heat shock protein 70; R = Reverse primer.

### Statistical analysis

Statistical analyses were performed using IBM SPSS Statistics version 25.0 (IBM Corp., Armonk, NY, USA). Data are presented as the mean ± standard deviation (SD). Data normality was assessed using the Shapiro–Wilk test, whereas homogeneity of variance was evaluated using Levene’s test.

Differences among treatment groups were assessed separately for each predefined endpoint using one-way analysis of variance (ANOVA). When the assumption of homogeneity of variance was satisfied, Tukey’s honestly significant difference post hoc test was applied. When variances were heterogeneous, the Games–Howell post hoc test was used. Specifically, *HSP70* expression and MMP were evaluated using Games–Howell post hoc comparisons, whereas sperm viability and MDA were evaluated using Tukey’s post hoc comparisons.

No additional adjustment for multiple comparisons across the four biologically distinct endpoints was applied. Consequently, cross-endpoint findings were interpreted with caution and evaluated based on biological consistency rather than a single global family-wise statistical test. Statistical significance was set at p < 0.05.

## RESULTS

### RNA quality control and *HSP70* primer validation

RNA was successfully extracted from frozen–thawed goat spermatozoa in all treatment groups. RNA concentrations ranged from 17.15 to 45.84 ng/µL, and the absorbance ratios at 260 and 280 nm (A260/A280) ranged from 1.842 to 1.983 (Table 3). These values were considered acceptable for subsequent reverse-transcription and amplification. Variation in RNA yield was not interpreted as a treatment effect because qPCR data were normalized using Ct-based relative expression. RIN values and 28S/18S ratios were unavailable.

Primer validation by 2% agarose gel electrophoresis revealed a single amplicon at the expected size of 167 bp, confirming the specificity of the *HSP70* primers prior to RT-qPCR analysis (Figure 1).

**Table 3:** RNA concentration and purity of post-thaw goat spermatozoa.

**Group (n = 8)**	**RNA concentration (ng/µL)**	**Purity (A260/A280)**
C0	17.15	1.887
C1	23.28	1.886
C2	45.84	1.921
C3	27.51	1.983
C4	40.33	1.842

Note: C0 = Vehicle-matched control without CoQ10; C1 = 5 mg/dL CoQ10; C2 = 10 mg/dL CoQ10; C3 = 20 mg/dL CoQ10; C4 = 40 mg/dL CoQ10; CoQ10 = Coenzyme Q10; RNA = Ribonucleic acid.

### Effects of CoQ10 on post-thaw sperm parameters

**Figure 2:**
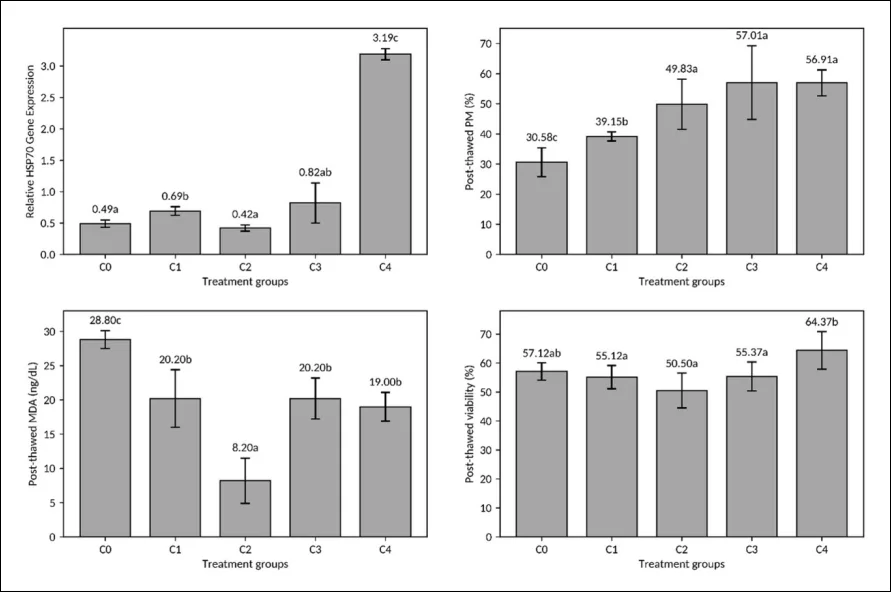
Effects of CoQ10 supplementation on post-thaw *HSP70* expression, MMP, MDA concentration, and viability of cryopreserved goat spermatozoa. Values are presented as mean ± SD (n = 8). Different superscript letters indicate significant differences among treatment groups within each endpoint (p < 0.05). CoQ10 = Coenzyme Q10; *HSP70* = Heat shock protein 70; MDA = Malondialdehyde; MMP = Mitochondrial membrane potential; SD = Standard deviation.

CoQ10 supplementation significantly affected *HSP70* transcript abundance, MMP, MDA concentration, and sperm viability (p < 0.001; Figure 2 and Table 4). The treatment response was non-linear: C4 produced the highest *HSP70* expression and sperm viability, whereas C2 produced the lowest MDA concentration. MMP was significantly greater in C2–C4 than in the untreated control.

**Relative HSP70 transcript abundance:** Relative *HSP70* expression differed significantly among the treatment groups (p < 0.001; Figure 2A and Table 4). C4 exhibited the highest expression, significantly higher than in all other groups (p < 0.05). Expression in C4 was approximately 6.5-fold higher than that in C0. C1 also exhibited significantly higher expression than C0 and C2 (p < 0.05), whereas C2 and C3 did not differ significantly from C0.

**Figure 3:**
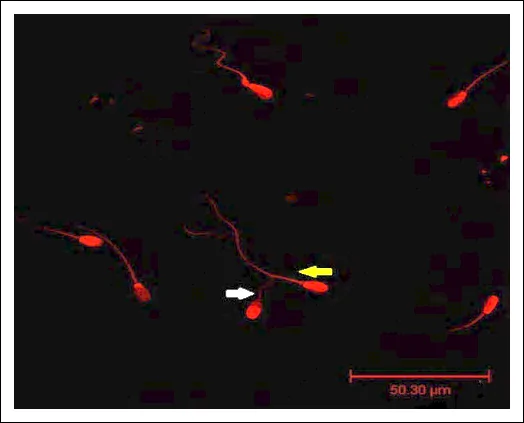
Representative confocal fluorescence micrograph of post-thaw goat spermatozoa stained with rhodamine 123, showing MMP in the neck and midpiece regions. Images were acquired using a Leica DMi8 STELLARIS 5 confocal microscope at 1000× magnification. White arrows indicate spermatozoa with weak fluorescence intensity and reduced MMP, whereas yellow arrows indicate spermatozoa with strong fluorescence intensity and high MMP. Scale bar = 50.30 µm. MMP = Mitochondrial membrane potential.

**MMP:** The proportion of spermatozoa with high MMP differed significantly among the treatment groups (p < 0.001; Figure 2B and Table 4). C0 exhibited the lowest MMP, which was significantly lower than that of all CoQ10-supplemented groups. C1 remained significantly lower than C2–C4, whereas no significant differences were detected among C2, C3, and C4. Representative rhodamine 123 fluorescence images illustrating spermatozoa with high and low MMP are presented in Figure 3.

**Table 4:** Post-thaw characteristics of cryopreserved goat spermatozoa supplemented with CoQ10.

**Group**	***HSP70*** ** relative expression**	**High MMP (%)**	**MDA (ng/µL)**	**Viability (%)**
C0	0.49 ± 0.06ᵃ	30.58 ± 4.83ᶜ	28.80 ± 1.38ᶜ	57.12 ± 3.13ᵃᵇ
C1	0.69 ± 0.06ᵇ	39.15 ± 1.55ᵇ	20.20 ± 4.20ᵇ	55.12 ± 4.22ᵃ
C2	0.42 ± 0.04ᵃ	49.83 ± 8.18ᵃ	8.20 ± 3.27ᵃ	50.50 ± 6.34ᵃ
C3	0.82 ± 0.32ᵃᵇ	57.01 ± 12.57ᵃ	20.20 ± 3.03ᵇ	55.37 ± 5.20ᵃ
C4	3.19 ± 0.08ᶜ	56.91 ± 4.21ᵃ	19.00 ± 2.23ᵇ	64.37 ± 6.58ᵇ

Note: Values are presented as mean ± SD. Different superscript letters within the same column indicate significant differences among treatment groups (p < 0.05). C0 = Vehicle-matched control without CoQ10; C1 = 5 mg/dL CoQ10; C2 = 10 mg/dL CoQ10; C3 = 20 mg/dL CoQ10; C4 = 40 mg/dL CoQ10; CoQ10 = Coenzyme Q10; *HSP70* = Heat shock protein 70; MDA = Malondialdehyde; MMP = Mitochondrial membrane potential; SD = Standard deviation.

**Lipid peroxidation:** Post-thaw MDA concentrations differed significantly among the treatment groups (p < 0.001; Figure 2C and Table 4). C0 exhibited the highest MDA concentration and was significantly higher than all CoQ10-supplemented groups. C2 exhibited the lowest MDA concentration, which was significantly lower than those of C0, C1, C3, and C4. Compared with C0, supplementation with 10 mg/dL CoQ10 reduced MDA by approximately 71.5%, indicating that suppression of lipid peroxidation was greatest at the moderate dose rather than at the highest dose.

**Figure 4:**
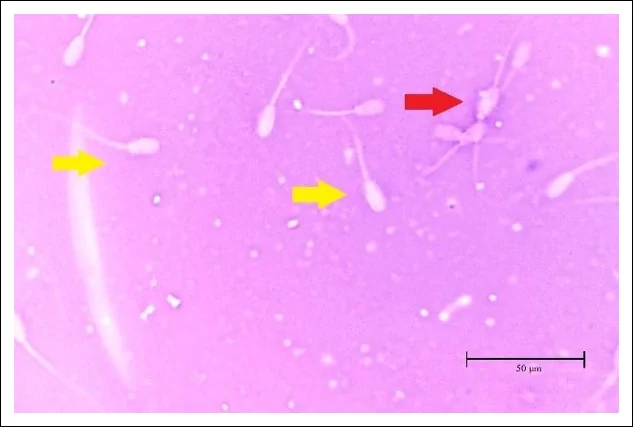
Representative post-thaw sperm viability assessment using eosin–nigrosine staining. Viable spermatozoa appear unstained or white, whereas nonviable spermatozoa appear pink or red. Magnification = 1000×; scale bar = 50 µm.

**Sperm viability:** Post-thaw sperm viability differed significantly among the treatment groups (p < 0.001; Figure 2D and Table 4). C4 exhibited the highest viability, significantly higher than C1, C2, and C3. C0 exhibited an intermediate value and did not differ significantly from C4 or C1–C3. Representative eosin–nigrosine-stained spermatozoa are shown in Figure 4.

### Descriptive relationship among endpoints

Across the treatment means, C4 exhibited the highest *HSP70* expression and sperm viability together with high MMP, whereas C2 exhibited the lowest MDA concentration. Individual-level correlation analysis and dose–response modeling were not prespecified, and inferential correlation coefficients were therefore not calculated. Consequently, the relationships among endpoints were interpreted descriptively. The observed pattern suggests that improved sperm viability was more closely associated with preservation of MMP and increased *HSP70* expression than with maximal suppression of MDA alone.

## DISCUSSION

### Overall effects of CoQ10 supplementation

The present study demonstrated that CoQ10 supplementation in Extender A modulated several post-thaw characteristics of PE goat spermatozoa, including *HSP70* expression, MMP, MDA concentration, and viability. The principal finding was a non-linear dose–response. C4 (40 mg/dL) produced the highest *HSP70* transcript abundance, maintained high MMP, and yielded the highest sperm viability, whereas C2 (10 mg/dL) produced the lowest MDA concentration. This divergence indicates that post-thaw sperm survival is not determined by a single oxidative stress marker. Rather, sperm survival appears to depend on the integrated preservation of mitochondrial function, plasma membrane integrity, and cellular stress-response capacity.

The simultaneous increase in *HSP70* expression, MMP, and viability in C4 suggests that the highest CoQ10 concentration tested may support both mitochondrial preservation and cellular adaptation to cryopreservation-induced stress. However, HSP70 protein abundance and its direct functional contribution were not evaluated. Therefore, the findings demonstrate an association among CoQ10 supplementation, *HSP70* expression, mitochondrial function, and sperm viability, but they do not establish that HSP70 directly mediated the observed cryoprotective effects.

### Comparison with previous studies

The beneficial effects observed in the present study are consistent with previous reports showing that CoQ10 and other antioxidants can improve sperm tolerance to cryopreservation in several animal species. Oktanella *et al.* [23] reported that CoQ10 supplementation in goat semen modulated *ATP5F1A* and *CPT2* expression and improved post-thaw cellular characteristics. Similarly, Khazravi *et al.* [24] found that CoQ10 supplementation helped preserve post-thaw buck sperm quality in a plant-based extender. Studies conducted in other species have also demonstrated that antioxidant supplementation can protect plasma membrane integrity and maintain sperm function during cooling and freezing [27].

Nevertheless, the effectiveness of antioxidant supplementation depends strongly on dose. Gardela *et al.* [28] reported that excessive CoQ10 supplementation may adversely affect rabbit semen, supporting the concept that redox-active antioxidants can exert biphasic or non-linear biological effects. The findings of the present study reinforce this concept because the CoQ10 concentration that most effectively reduced MDA was not the same concentration that produced the highest viability and *HSP70* expression. Therefore, extender optimization should not be based solely on a single oxidative stress biomarker but should incorporate multiple functional, biochemical, and molecular endpoints.

### Mitochondrial protection and oxidative status

Mitochondria are particularly vulnerable to freeze–thaw injury because disruption of mitochondrial membranes and electron transport can increase ROS generation while reducing ATP availability [29–32]. The higher proportion of spermatozoa with high MMP in C2–C4, particularly C3 and C4, indicates improved preservation of mitochondrial electrochemical function after thawing. CoQ10 may contribute to this protection through its role in mitochondrial electron transport and its ability to limit oxidative damage in lipid-rich mitochondrial membranes [28, 33].

However, mitochondrial respiration, ATP production, electron transport-chain activity, voltage-dependent anion channel (VDAC) function, and mitochondrial permeability transition pore (mPTP) opening were not directly evaluated. Consequently, interpretations involving these pathways should be regarded as biologically plausible explanations supported by previous literature rather than as mechanisms demonstrated by the present experiment.

The MDA findings also caution against assuming that increasing antioxidant concentrations will continuously reduce lipid peroxidation. C2 produced the lowest MDA concentration, whereas C3 and C4 maintained MDA concentrations below that of C0 but not as low as that of C2. One possible explanation is that a moderate CoQ10 concentration may be optimal for suppressing overall lipid peroxidation, whereas higher concentrations may preferentially support mitochondrial electron transport, redox cycling, and bioenergetic continuity. Alternatively, high concentrations of redox-active quinones may exert localized pro-oxidant effects under specific experimental conditions, as reported in other biological systems [28, 33].

MDA represents only one component of oxidative damage and does not fully capture mitochondrial ROS microdomains, oxidative protein modifications, or aldehyde–protein adduct formation [34, 35]. In addition, the thiobarbituric acid-reactive substances (TBARS) assay is an indirect indicator of lipid peroxidation and may not capture all oxidative processes occurring within post-thaw spermatozoa. Therefore, the higher viability observed in C4, despite its higher MDA concentration than in C2, may reflect superior maintenance of MMP and cellular stress adaptation rather than maximal suppression of the TBARS-derived MDA signal alone.

### HSP70 expression and post-thaw viability

HSP70 is a molecular chaperone that contributes to protein stabilization, refolding of damaged proteins, and prevention of protein aggregation under thermal and oxidative stress [14, 15, 36]. Previous studies have associated members of the HSP family with sperm freezability, cryotolerance, and post-thaw quality [16, 17, 37]. In the present study, C4 exhibited the highest *HSP70* transcript abundance and the highest viability and MMP. This coordinated pattern suggests that supplementation with CoQ10 to 40 mg/dL may be associated with a more pronounced stress-response phenotype in cryopreserved PE goat spermatozoa.

Nevertheless, mature spermatozoa possess limited transcriptional and translational capacities, and an increase in messenger RNA abundance does not necessarily indicate a corresponding increase in protein abundance or chaperone activity. Furthermore, the present study did not determine whether the detected transcripts were newly regulated during processing or represented differential preservation of pre-existing sperm RNA. Western blotting, immunofluorescence, proteomic analysis, HSP70 inhibition, and other functional assays would be required to determine whether HSP70 protein directly contributes to mitochondrial protection and post-thaw survival.

Eosin–nigrosine staining assesses plasma membrane permeability rather than mitochondrial function directly. However, maintenance of plasma membrane integrity after thawing depends on effective ionic regulation, membrane stabilization, and cellular energy availability, all of which are influenced partly by mitochondrial integrity [38–42]. Therefore, the higher viability observed in C4 is biologically consistent with the high MMP recorded in this group.

Conversely, C2 exhibited the lowest MDA concentration but did not produce the highest viability. This finding indicates that suppression of lipid peroxidation alone may be insufficient to preserve all post-thaw sperm functions. It also highlights the importance of simultaneously assessing mitochondrial function, oxidative damage, membrane integrity, and molecular stress responses when optimizing semen cryopreservation extenders.

### Proposed CoQ10–HSP70–mitochondrial protective pathway

**Figure 5:**
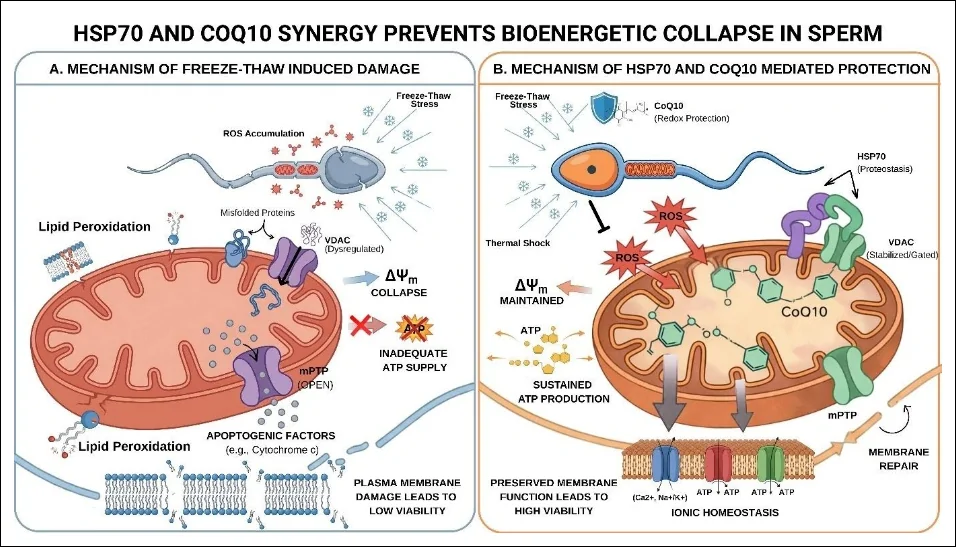
Conceptual model of freeze–thaw-induced sperm damage and the proposed associations among CoQ10 supplementation, the HSP70 stress-response, and mitochondrial protection. The schematic presents a working hypothesis derived from the endpoints evaluated in this study and previously published mitochondrial stress literature. Direct VDAC activity, mPTP opening, cytochrome c release, caspase activation, and HSP70 protein abundance were not evaluated. CoQ10 = Coenzyme Q10; HSP70 = Heat shock protein 70; MMP = Mitochondrial membrane potential; mPTP = Mitochondrial permeability transition pore; ROS = Reactive oxygen species; VDAC = Voltage-dependent anion channel. Source: Authors’ original illustration, conceptually developed based on previous studies [14, 17, 23, 24, 30–32, 34].

Figure 5 presents a conceptual model rather than a directly proven biological pathway. During freeze–thaw stress, excessive ROS generation and lipid peroxidation may destabilize mitochondrial membranes and contribute to mitochondrial permeability dysregulation [43, 44]. CoQ10 may reduce oxidative pressure on mitochondrial membranes while supporting electron transport, whereas HSP70 may help stabilize proteins and preserve their conformation during cryopreservation-induced stress [33, 36, 45]. Together, these processes could help maintain MMP, membrane integrity, and post-thaw sperm viability.

However, VDAC activity, mPTP opening, cytochrome c release, caspase activation, DNA fragmentation, and HSP70 protein abundance were not measured. Therefore, the proposed CoQ10–HSP70–mitochondrial axis should be regarded as a working hypothesis that requires functional validation.

### Study limitations

Several limitations should be considered when interpreting the findings. First, all ejaculates were obtained from a single PE buck. Although the split-ejaculate design reduced variation among treatments within each ejaculate, it could not account for biological variability among males. Consequently, the results should be considered exploratory and not generalized to the broader PE goat population without validation with semen from multiple bucks.

Second, the study evaluated a limited set of post-thaw laboratory endpoints. Post-thaw motility assessed by CASA, acrosome integrity, DNA fragmentation, intracellular ROS production, apoptotic markers, mitochondrial respiration, ATP concentration, and *in vivo* fertility were not evaluated. Third, only *HSP70* transcript abundance was measured. HSP70 protein abundance, localization, and functional activity were not assessed, preventing confirmation of a direct mediating role.

Fourth, rhodamine 123 staining provided a comparative assessment of MMP because chemical depolarization controls and flow-cytometric quantification of fluorescence intensity were not included. Fifth, extender pH and osmolarity, CoQ10 solubility and stability by HPLC, qPCR primer efficiencies, reference gene stability, and RNA integrity were not formally assessed. These limitations do not invalidate the observed treatment differences, but they restrict the strength of mechanistic interpretation and the immediate application of the findings to commercial semen production.

### Future research

Future studies should evaluate CoQ10 concentrations within the 10–40 mg/dL range using semen collected from multiple PE bucks and a larger number of ejaculates. These investigations should include treatment-specific pre-freeze and post-thaw motility measured by CASA, acrosome and plasma membrane integrity, DNA fragmentation, intracellular and mitochondrial ROS profiling, mitochondrial respiration, ATP production, and apoptotic markers.

Further molecular studies should quantify HSP70 protein abundance and localization and assess whether pharmacological or molecular inhibition of HSP70 alters the protective effects of CoQ10. Validation of multiple reference genes, determination of qPCR amplification efficiencies, and assessment of sperm RNA integrity would also strengthen transcript-level interpretation. In addition, extender pH, osmolarity, CoQ10 solubility, and stability should be characterized to ensure consistent formulation and reproducibility.

Most importantly, fertility trials following AI are required to determine whether the *in vitro* advantages observed with 40 mg/dL CoQ10 translate into improved conception, pregnancy, kidding, and offspring outcomes. Such trials should also determine whether the marked reduction in MDA at 10 mg/dL has practical relevance to field fertility, sperm longevity in the female reproductive tract, or long-term semen storage.

## CONCLUSION

The present study demonstrated that supplementation of Extender A with CoQ10 significantly influenced the post-thaw quality of cryopreserved PE goat spermatozoa. A clear dose-dependent but non-linear response was observed. Supplementation with 40 mg/dL CoQ10 produced the greatest *HSP70* transcript abundance, maintained high MMP, and resulted in the highest post-thaw sperm viability, whereas supplementation with 10 mg/dL CoQ10 produced the lowest MDA concentration, indicating the strongest suppression of lipid peroxidation. These findings suggest that optimal post-thaw sperm survival depends on the coordinated preservation of mitochondrial function, cellular stress responses, and membrane integrity, rather than on the maximal reduction of oxidative stress alone.

From a practical perspective, incorporation of CoQ10 into Extender A represents a simple and potentially effective modification to conventional goat semen cryopreservation protocols. Early antioxidant supplementation during semen dilution and cooling may improve post-thaw sperm quality by preserving mitochondrial function and enhancing cellular resistance to cryoinjury. If confirmed in subsequent fertility studies, this strategy could improve the efficiency of AI programs, facilitate dissemination of superior genetics, and contribute to sustainable genetic improvement of PE goats and other caprine breeds.

A major strength of this study is the comprehensive evaluation of cryopreservation outcomes by integrating molecular (*HSP70* expression), functional (MMP and sperm viability), and biochemical (MDA) endpoints within a split-ejaculate experimental design, thereby reducing within-ejaculate variability and providing a more comprehensive assessment of CoQ10-mediated cryoprotection than conventional semen-quality evaluations alone.

Overall, the findings indicate that supplementing Extender A with 40 mg/dL CoQ10 yields the greatest benefit in preserving post-thaw PE goat sperm quality, although 10 mg/dL CoQ10 reduces lipid peroxidation the most. These results provide new molecular and functional evidence supporting the use of CoQ10 as a promising cryoprotective additive and establish a foundation for optimizing semen cryopreservation protocols to improve the success of caprine AI programs.

## DATA AVAILABILITY

The data generated during the study are included in the manuscript.

## GENERATIVE AI DECLARATION

The authors declare that generative artificial intelligence (AI) tools were used to improve language, grammar, and readability during manuscript preparation. The authors also used generative artificial intelligence tools to assist with language editing and the preparation of the conceptual illustration presented in Figure 5. All scientific content, data analysis, interpretation of results, and conclusions were developed and verified by the authors. The authors take full responsibility for the accuracy, integrity, and originality of the work presented, and no AI tool was listed as an author.

## AUTHORS’ CONTRIBUTIONS

YO and SS: project administration, and writing—original draft preparation. IM and TWS: Conceptualization, study supervision, and writing—review and editing. YO and WW: Formal analysis, methodology, and writing—review and editing. YK: Sample collection, methodological support, statistical analysis, and writing—review and editing. All authors have read and approved the final version of the manuscript.
